# Human milk oligosaccharides modulate the intestinal microbiome of healthy adults

**DOI:** 10.1038/s41598-023-41040-5

**Published:** 2023-08-31

**Authors:** Jonathan P. Jacobs, Martin L. Lee, David J. Rechtman, Adam K. Sun, Chloe Autran, Victoria Niklas

**Affiliations:** 1 The Vatche and Tamar Manoukian Division of Digestive Diseases, Department of Medicine, David Geffen School of Medicine at UCLA, Los Angeles, CA USA; 2 Goodman-Luskin Microbiome Center, University of California Los Angeles, Los Angeles, CA USA; 3 Fielding School of Public Health, University of California Los Angeles, Los Angeles, CA USA; 4 Prolacta Bioscience, Duarte, CA USA; 5 Department of Pediatrics, David Geffen School of Medicine at UCLA, Los Angeles, CA USA; 6 Oak Hill Bio Ltd, Altrincham, Cheshire WA14 2DT, United Kingdom

**Keywords:** Microbiome, Microbiota

## Abstract

Human milk contains over 200 distinct oligosaccharides, which are critical to shaping the developing neonatal gut microbiome. To investigate whether a complex mixture of human milk oligosaccharides (HMOs) would similarly modulate the adult gut microbiome, HMO-Concentrate derived from pooled donor breast milk was administered orally to 32 healthy adults for 7 days followed by 21 days of monitoring. Fecal samples were collected for 16S rRNA gene sequencing, shotgun metagenomics, and metabolomics analyses. HMO-Concentrate induced dose-dependent *Bifidobacterium* expansion, reduced microbial diversity, and altered microbial gene content. Following HMO cessation, a microbial succession occurred with diverse taxonomic changes—including *Bacteroides* expansion—that persisted through day 28. This was associated with altered microbial gene content, shifts in serum metabolite levels, and increased circulating TGFβ and IL-10. Incubation of cultured adult microbiota with HMO-Concentrate induced dose-dependent compositional shifts that were not recapitulated by individual HMOs or defined mixtures of the 10 most abundant HMOs in HMO-Concentrate at their measured concentrations. These findings support that pooled donor HMOs can exert direct effects on adult gut microbiota and that complex mixtures including low abundance HMOs present in donor milk may be required for maximum effect.

*Registration*: ClinicalTrials.gov NCT05516225

## Introduction

Breast milk contains high concentrations of structurally diverse oligosaccharides that together constitute the third most abundant component in breast milk, following lactose and fat^1–3^. As many as 200 different HMO structures varying from 3 to 22 sugar molecules have been identified in human breast milk^4^. HMOs consist of a core lactose backbone with fucose, sialic acid or N-acetyl glucosamine side chains that, in part, determine their specific biological functions^4–7^. HMOs are not metabolized as an energy source by the infant and the majority are resistant to hydrolysis in the stomach and upper gastrointestinal tract, allowing their passage intact to the colon^8–11^.

HMOs have diverse effects in the neonatal gut including promoting the growth of specific colonic bacteria, interfering with microbial adhesion to the intestinal lining, binding to bacterial toxins, and shaping epithelial and mucosal immune development^12–15^. Particular commensal bacteria in infants known to utilize HMOs as an energy source include *Bifidobacterium* as well as *Bacteroides* and *Roseburia* species^2,16,17^. Metabolism of HMOs by intestinal microbiota has been demonstrated in vitro and has been inferred in infants by the presence of HMO degradation products in feces^11,18–21^. Concordant with these findings, human microbiome association studies have consistently reported distinct microbiome profiles in breastfed infants compared to formula-fed infants—in particular expansion of *Bidifobacterium*—as well as associations between HMO concentrations in breast milk and infant gut microbiome composition^22–28^. Fermentation of HMOs by commensal gut microbes such as *Bifidobacterium* results in the production of short-chain fatty acids (SCFA), which improve intestinal barrier function and have well established anti-inflammatory properties^29–31^.

Due to the benefits of HMOs and their high abundance in breast milk, there has been considerable interest in including specific HMOs in commercial formulations for infant nutrition. However, modulation of gut microbiota by HMOs also has the potential to restore health-associated microbial communities in adult diseases such as obesity, diabetes, and inflammatory bowel disease which have been linked to dysbiosis (i.e. alterations of gut microbiome composition and function). In vitro studies of cultured human microbiota have demonstrated that two specific HMOs—2’-O-fucosyllactose (2’FL) and lacto-N-neotetraose (LNnT) – could promote *Bifidobacterium* and SCFA production^32^. In a proof of concept interventional study, 2’FL and LNnT were administered alone or in combination to healthy adult volunteers for two weeks^33^. The authors reported that these specific HMOs were well-tolerated and associated with changes in fecal microbiota composition including expansion of *Bifidobacterium*. A second study reported that adults with irritable bowel syndrome receiving a combination of 2’FL and LNnT for four weeks showed changes in fecal microbial composition, including increased *Bifidobacterium*, and in fecal and plasma metabolites^34^. Complex mixtures of HMOs may have distinct effects on the gut microbiome compared to individual HMOs given the structural diversity among HMOs and prior reports that individual HMOs vary in their effects on cultured bacterial strains^35–38^. To investigate the potential for a HMO mixture reflecting the full complexity of human breast milk to modulate the adult gut microbiome, we conducted a clinical study in healthy adults to evaluate the effects of a concentrated HMO formulation derived from human donor breast milk on intestinal microbiome composition/function using a multi-omics strategy incorporating 16S rRNA gene sequencing, shotgun metagenomics, metabolomics, and serum cytokine measurement. Microbiome shifts induced by the concentrated HMO formulation were compared to those of individual HMOs in anaerobic cultures of human feces.

## Results

### Study demographics and safety

**Figure 1:**
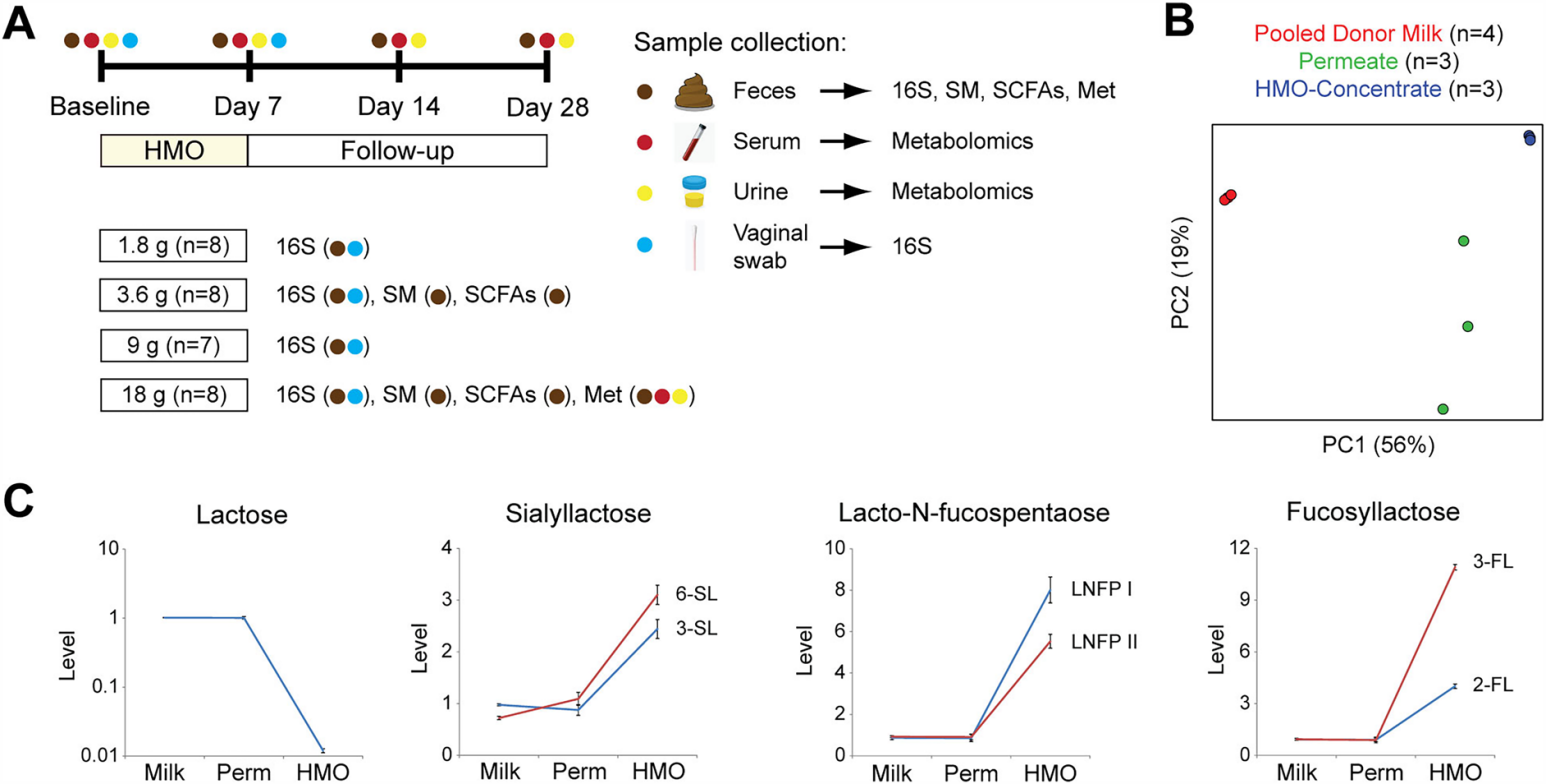
Study design overview. (**A**) Diagram depicting allocation of participants to four HMO dose cohorts, each receiving HMO-Concentrate for 7 days followed by 21 days of longitudinal sampling of feces, serum, urine, and vaginal swabs. Assays performed on each sample type are shown by dose cohort. 16S = 16S rRNA gene sequencing, SM = shotgun metagenomics, SCFAs = short chain fatty acids, Met = metabolomics (**B**) Global metabolomics comparison of pooled donor milk, permeate, and HMO-Concentrate. (**C**) Levels of lactose and representative examples of sialylated and fucosylated oligosaccharides in HMO-Concentrate. Data were scaled such that the median of all samples was 1.

Thirty-two healthy volunteers were recruited with 8 assigned to each of four dose groups (1.8 g, 3.6 g, 9 g, and 18 g daily) receiving HMO-Concentrate daily for 7 days followed by 21 days of follow-up (Fig. 1A). The HMO-Concentrate was derived by removing lactose from permeate generated during the production of protein-enriched nutritional fortifiers from pooled donor human breast milk. It was confirmed by metabolomics to be enriched in HMOs and depleted of lactose compared to the original donor breast milk and permeate (Fig. 1B, C). The mean age was 31 years (range 22–45 years) and mean BMI was 22.5 kg/m^2^ (range 18.5–24.9 kg/m^2^). Nineteen of the subjects were Caucasian (60%), ten were African-American (31%), two were mixed race (6%), and one was Asian (3%). The majority of subjects were female (63%) (Table S1). Of the 32 subjects who enrolled in this study, 30 completed the study. One dropped out after baseline sampling before completing HMO treatment and was excluded from all except safety analyses. A second withdrew after sampling at day 14 and was included in all analyses except those requiring day 28 outcomes.

No adverse events were reported for any of the 32 subjects. ECG readings were normal and there were no significant differences in heart rate, respiratory rate, blood pressure, temperature, electrolytes, renal function, liver function, complete blood counts, and urinalyses following exposure to the HMO-Concentrate (data not shown). All but two subjects had fecal calprotectin < 50 µg/g at baseline and there were no statistically significant changes in fecal calprotectin during the study (data not shown).

### HMO-concentrate reduces microbial diversity and alters microbiome composition

**Figure 2:**
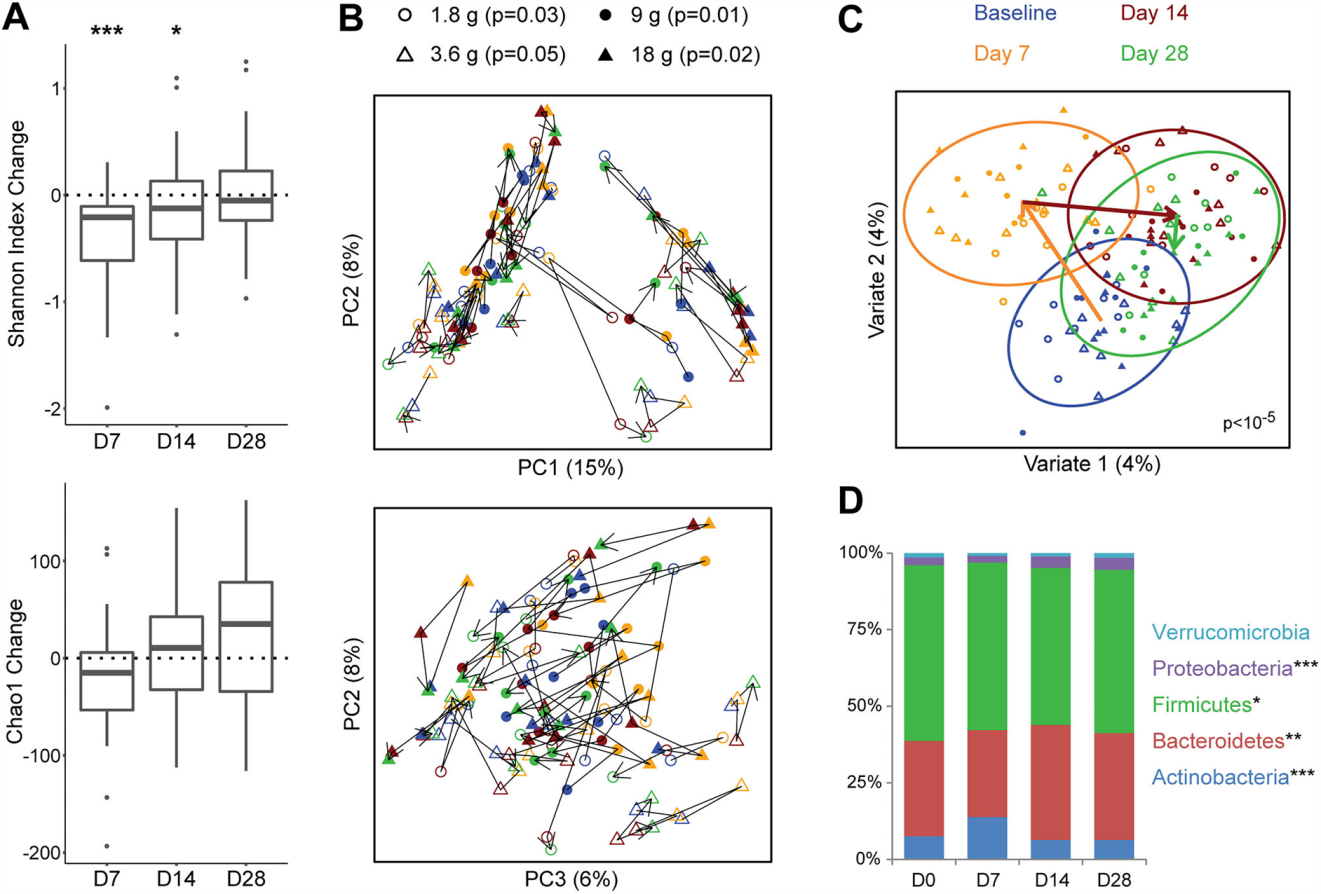
HMO administration alters gut microbiome composition and diversity. (**A**) Boxplots show changes in alpha diversity (measured by Chao1 and Shannon index) from baseline at three time points: day 7 (D7), day 14 (D14), and day 28 (D28). Boxes represent the first quartile, median, and third quartile. Whiskers extend to 1.5 times the interquartile range and outlying points are individually plotted. *p < 0.05, **p < 0.01, ***p < 0.001 (**B**) PCoA plots depicting microbiome composition across the four time points. The two plots show the first three principal coordinates and the percent variation that they explain. Each dot represents one sample, with lines connecting samples collected from each subject. Color represents time point and symbol represents dose group. *P*-values for each dose group were calculated by Adonis adjusting for subject. (**C**) Multilevel sPLS-DA plot visualizing shifts in microbiome composition across the four time points controlling for subject. Ellipses indicate the 95% confidence region for each time point. Arrows indicate change in centroids at each time point. *P*-value was calculated by Adonis for all doses combined, adjusting for subject. (**D**) Mean relative abundance at the phylum level for all doses combined. *p < 0.05, **p < 0.01, ***p < 0.00001 across all time points.

The effects of HMO-Concentrate on intestinal microbiome composition were assessed by 16S rRNA gene sequencing of stool collected at baseline and days 7, 14, and 28. Microbial diversity within samples (alpha diversity) as measured by the Shannon index—which incorporates both microbial richness and evenness—showed highly significant differences across the four study time points (*p* = 0.0002) when all dose groups were combined. This was driven by significant decreases at Day 7 (*p* = 0.0001) and to a lesser extent Day 14 (*p* = 0.02) compared to baseline (Fig. 2A). Microbial richness alone (Chao1 index) showed a statistically significant change over the course of the study (*p* = 0.04), driven by a significant increase in richness at day 28 relative to day 7 (*p* = 0.009) and a trend towards reduced richness on day 7 compared to baseline (*p* = 0.1) (Fig. 2A).

Variation in intestinal microbiome composition across samples (beta diversity) was largely attributable to inter-individual differences, but highly significant shifts in microbial composition could be detected across the study time points when all dose groups were combined (*p* < 10^–5^) (Fig. 2B, C). This was driven by shifts in microbial composition on day 7 relative to baseline, evident as a rightward shift in PC3 of principal coordinates analysis (PCoA) (Fig. 2B). Multilevel sparse partial least squares discriminant analysis (sPLS-DA) was performed to visualize data after adjustment for inter-individual differences and demonstrated a subsequent shift in microbiome composition on day 14 relative to both day 7 and baseline; day 14 and day 28 were similar (Fig. 2C). At the phylum level, these changes corresponded to an increased proportion of Actinobacteria on day 7, which subsequently returned to baseline; decreased Firmicutes on days 14 and 28; and increased Bacteroidetes and Proteobacteria on days 14 and 28 (Figs. 2D, S1).

The vaginal microbiome was assessed in female subjects at baseline and day 7. There was no significant change in vaginal microbial diversity at day 7 across all dose groups (Fig. S2A). There was also no change in overall vaginal microbial community structure after HMO treatment and no taxa were differentially abundant (Fig. S2B).

### HMO-concentrate induces *Bifidobacterium* expansion

**Figure 3:**
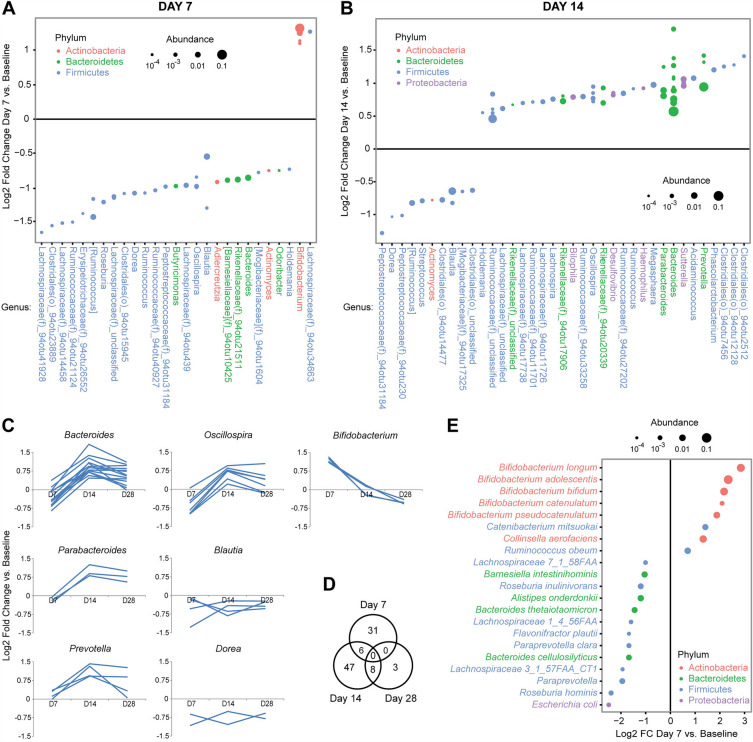
HMO-Concentrate induces an initial *Bifidobacterium* expansion followed by further microbiome change including *Bacteroides* expansion. (**A**) Differentially abundant OTUs at day 7 were identified from 16S rRNA gene sequencing data by DESeq2 models adjusting for subject. Each dot represents one OTU, which are organized by genus with color representing phylum and dot size proportional to normalized abundance. Effect size is shown as the log2 of the fold change. (**B**) Differentially abundant OTUs at day 14 relative to baseline are shown. (**C**) Kinetic profiles of differential OTUs for selected genera. (**D**) Venn diagram showing the number of differential OTUs relative to baseline at each time point. (**E**) Differentially abundant species identified by shotgun metagenomics at day 7.

Differential abundance testing identified 37 operational taxonomic units (OTUs) that were significantly altered by HMO on day 7 when considering all dose groups together (Fig. 3A). Of these, 5 were enriched which included 4 OTUs identified as *Bifidobacterium* species (within the Actinobacteria phylum) and one unclassified OTU in the Lachnospiraceae family. The remaining 31 differential OTUs that were depleted spanned a wide range of genera within the Firmicutes and Bacteroidetes phyla including *Oscillospira*, *Roseburia*, *Blautia*, *Ruminococcus*, and *Bacteroides*. This pattern of selective microbial expansion and depletion of a wider taxonomic range is consistent with the decreased microbial diversity on day 7.

**Figure 4:**
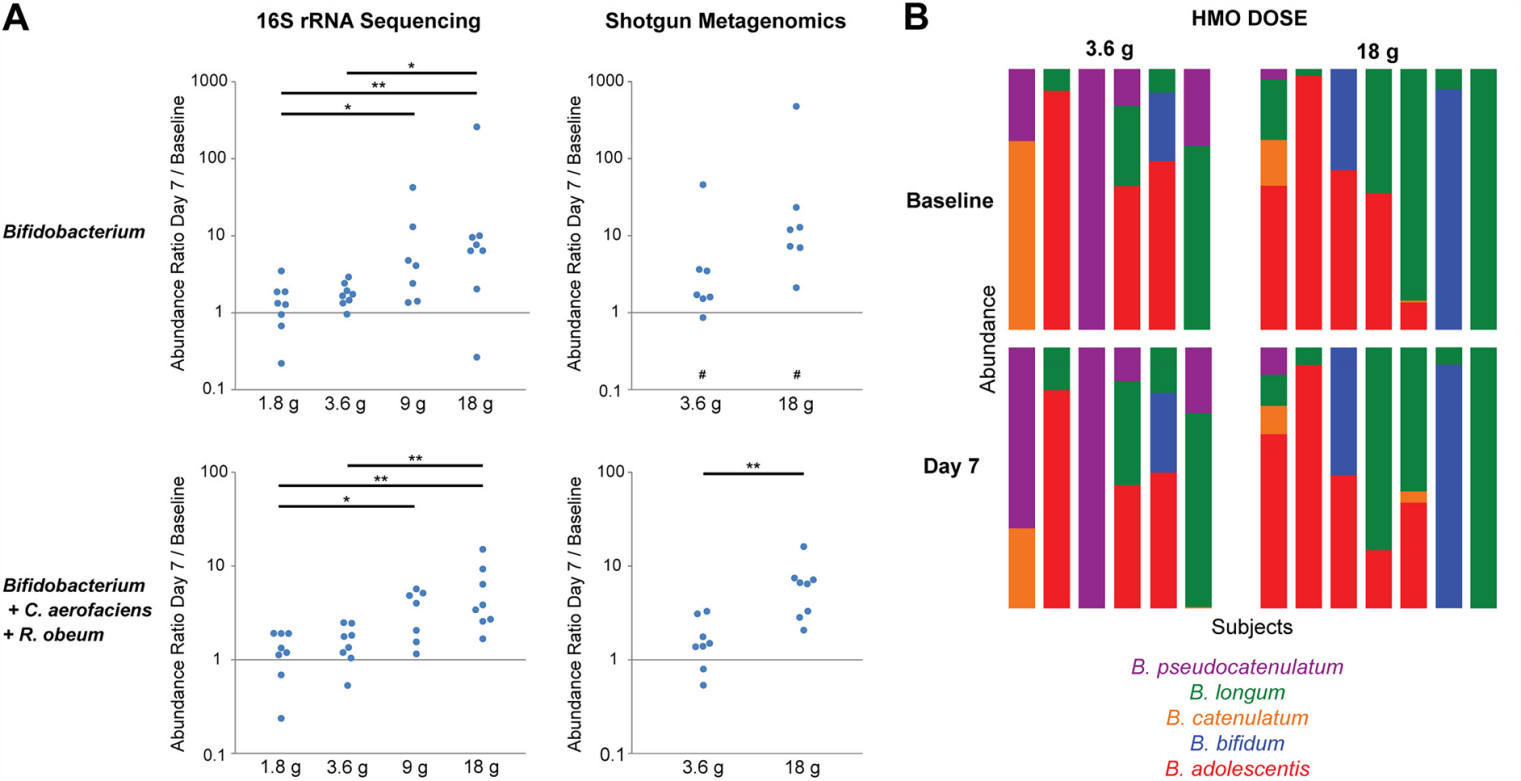
Dose-dependent expansion of intestinal *Bifidobacterium* without change in species distribution. (**A**) Graphs depict the ratio of *Bifidobacterium* relative abundance at day 7 compared to day 0 based upon 16S rRNA gene sequencing or shotgun metagenomics. Each dot represents one patient. The two lower panels show abundance ratios when adding counts of *Collinsella aerofaciens* and *Ruminococcus obeum* to *Bifidobacterium*. # signifies samples without detectable *Bifidobacterium*. *p < 0.05, **p < 0.005 (**B**) Stacked bar graphs represent the distribution of *Bifidobacterium* species in subjects in the 3.6 g and 18 g dose groups at baseline and at day 7 based upon shotgun metagenomics. Each of five detected *Bifidobacterium* species is represented by a different color. The colored area of each species is proportional to its relative abundance out of all detected *Bifidobacterium* in a subject. Two subjects with undetectable *Bifidobacterium* were not included.

Shotgun metagenomics sequencing was then performed on fecal samples from the 3.6 g and 18 g dose groups to provide improved species level resolution. This approach demonstrated enrichment of 5 *Bifidobacterium* species at day 7 after HMO treatment, including *B. adolescentis*, *B. longum*, *B. bifidum*, *B. catenulatum*, and *B. pseudocatenulatum* (Fig. 3E). Induction of *Bifidobacterium* was dose dependent, with subjects in the two highest dose groups showing a mean change from 3% *Bifidobacterium* abundance at baseline to 13% at day 7 with one subject reaching 33% abundance (Fig. 4A). There was no change in the distribution of *Bifidobacterium* species after HMO treatment, suggesting that HMO does not preferentially expand any of the five responding species in adults (Fig. 4B).

The shotgun metagenomics data also demonstrated enrichment of *Ruminococcus obeum* and *Collinsella aerofaciens*. *Ruminococcus obeum* has previously been reported to be a major constituent of the infant microbiome and is phylogenetically assigned to the Lachnospiraceae family, matching the unclassified Lachnospiraceae OTU enriched at day 7^39^. *Collinsella aerofaciens* is a member of the Actinobacteria phylum that expands in vitro in response to HMO^40^. Combining *R. obeum* and *C. aerofaciens* abundances with *Bifidobacterium* abundance resulted in a stronger HMO dose response relationship than was seen with *Bifidobacterium* alone (Fig. 4A). The shotgun metagenomics data also demonstrated depletion of 13 microbes including *Escherichia coli*, *Bacteroides thetaiotamicron*, and *Roseburia hominis*.

### Gut microbiome shifts occur after cessation of HMO treatment

Differential abundance testing was then performed for days 14 and 28 to assess for microbiome shifts during the time period following cessation of HMO treatment. 61 OTUs were differentially abundant at day 14, of which only 6 overlapped with those changes seen at the end of HMO treatment on day 7 including persistent depletion in members of the *Blautia* and *Dorea* genera (Fig. 3A–D). *Bifidobacterium* species were not significantly different from baseline by day 14 (Fig. 3C). The limited overlap is consistent with the finding that overall microbial composition on day 14 was distinct from both day 7 and baseline (Fig. 2C). The microbes enriched after HMO treatment included 13 members of the *Bacteroides* genus, which collectively showed a trend towards depletion on day 7 but clear expansion compared to baseline on day 14 (Fig. 3B–C). In addition, members of the *Parabacteroides* and *Prevotella* genera—which like *Bacteroides* are both within the Bacteriodales order—as well as a third genera, *Oscillospira*, were also expanded on day 14 (Fig. 3C).

Day 28 was characterized by 11 differentially abundant OTUs, of which 8 had been differentially abundant on day 14 in the same direction (Fig. 3D, data not shown). The reduced number of significant changes at this later time point reflected attenuation of the changes seen on day 14 for a number of genera including *Bacteroides*, *Parabacteroides*, *Prevotella*, and *Oscillospira* (Fig. 3C). No differentially abundant OTUs were identified at days 14 and 28 using the shotgun metagenomics data, which may have reflected reduced sample size (since only two dose groups underwent metagenomics analysis).

### HMO-concentrate modulates microbial function

**Figure 5:**
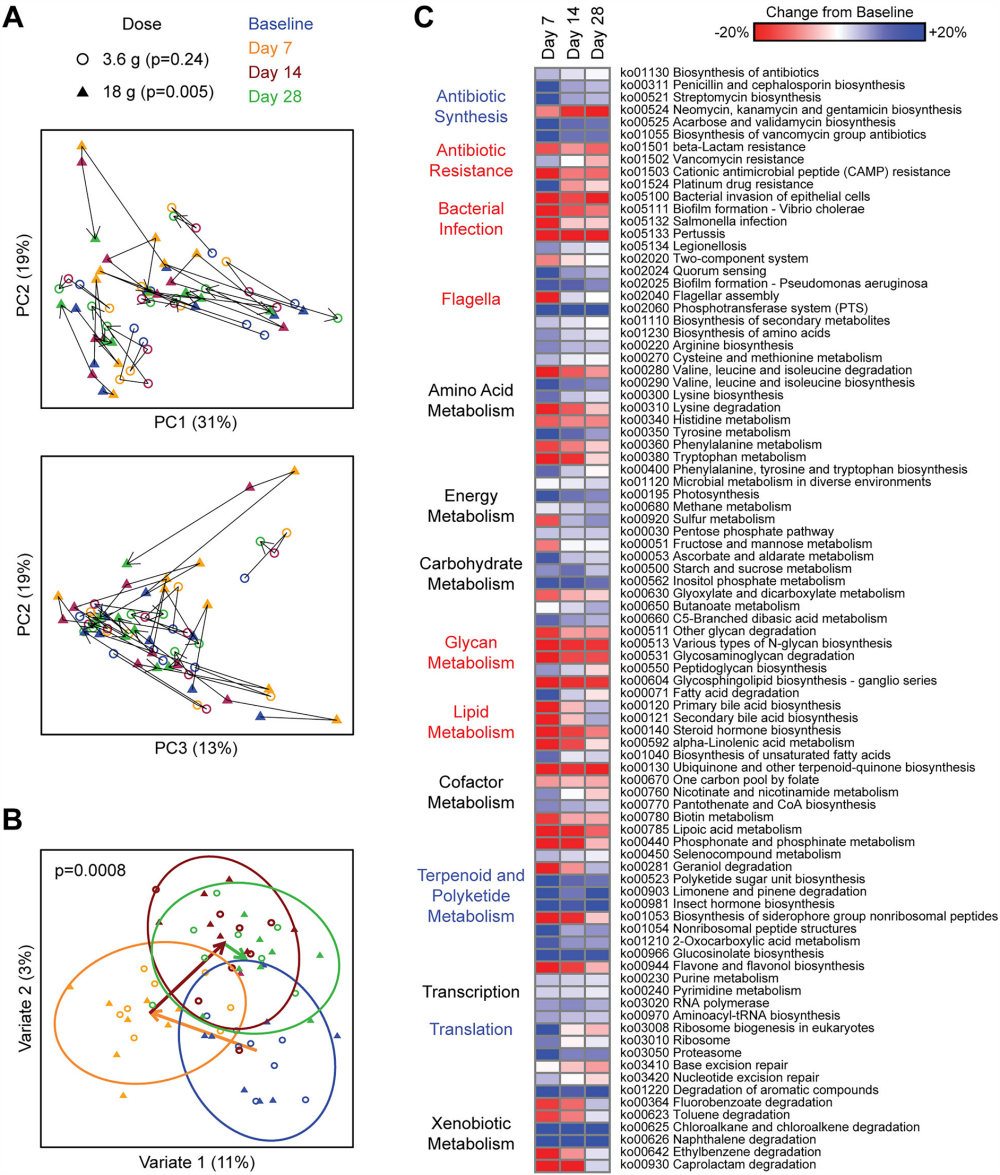
HMO treatment alters microbial gene content including induction of antibiotic synthesis pathways and depletion of antibiotic resistance pathways. (**A**) PCoA plots depicting microbial gene content by shotgun metagenomics across the four time points. Each dot represents one sample, with lines connecting samples collected from each subject. (**B**) Multilevel sPLS-DA plot visualizing shifts in microbial gene content across the four time points controlling for subject. Ellipses indicate the 95% confidence region for each time point. Arrows indicate change in centroids at each time point. P-value was calculated by Adonis for all doses combined, adjusting for subject. (**C**) Heat map showing differentially abundant microbial functional pathways (KEGG annotation) ordered by functional category. Color gradient indicates percentage increase or decrease in normalized gene counts belonging to each pathway. Category labels to the left of the heat map are colored if the mean change of differential pathways in that category was greater than 5% (blue) or less than -5% (red).

The functional consequences of gut microbiome alterations with HMO treatment were assessed by shotgun metagenomics analysis of the 3.6 g and 18 g dose groups. A highly significant change in bacterial gene content was seen over time when considering both dose groups (*p* = 0.0008) (Fig. 5A, B). The pattern mirrored that observed with microbial composition: a shift in gene content between baseline and day 7, followed by a transition to a distinct state by day 14 (distinct from baseline) which largely persisted on day 28 (Fig. 5B). Differential abundance testing at the KEGG pathway level demonstrated 89 differentially abundant functional pathways at day 7 belonging to diverse functional categories including metabolism (carbohydrates, amino acids, lipids), transcription, and translation (Fig. 5C). Notably, HMO intake was associated with increased levels of pathways involved in antibiotic synthesis and decreased levels of pathways mediating antibiotic resistance, biofilm formation, and bacterial invasion/infection. The direction of these changes largely persisted on days 14 and 28, though most were attenuated and lost significance with the exception of “bacterial invasion of epithelial cells” and “neomycin, kanamycin and gentamicin biosynthesis,” which retained significance and showed similar or greater effect size on day 28 as day 7.

The functional consequences of the microbiome shifts induced by HMO administration were further assessed by fecal metabolomics. Given the known capacity of *Bifidobacterium* to generate SCFAs from HMOs, we performed targeted measurement of fecal SCFAs in samples from the 3.6 g and 18 g dose groups. Short chain fatty acids showed wide variation across study time points, but there was a trend towards significant changes in acetate (*p* = 0.09) and butyrate (*p* = 0.08) driven by increased levels on day 7 (Fig. S3). Global metabolomics analysis was also performed for all fecal samples from the 18 g dose group. There was no significant shift in the overall fecal metabolome during the study and no individual fecal metabolites were differentially abundant across the four time points (Fig. S4).

### Systemic effects of HMO-concentrate on circulating metabolites and regulatory cytokines

**Figure 6:**
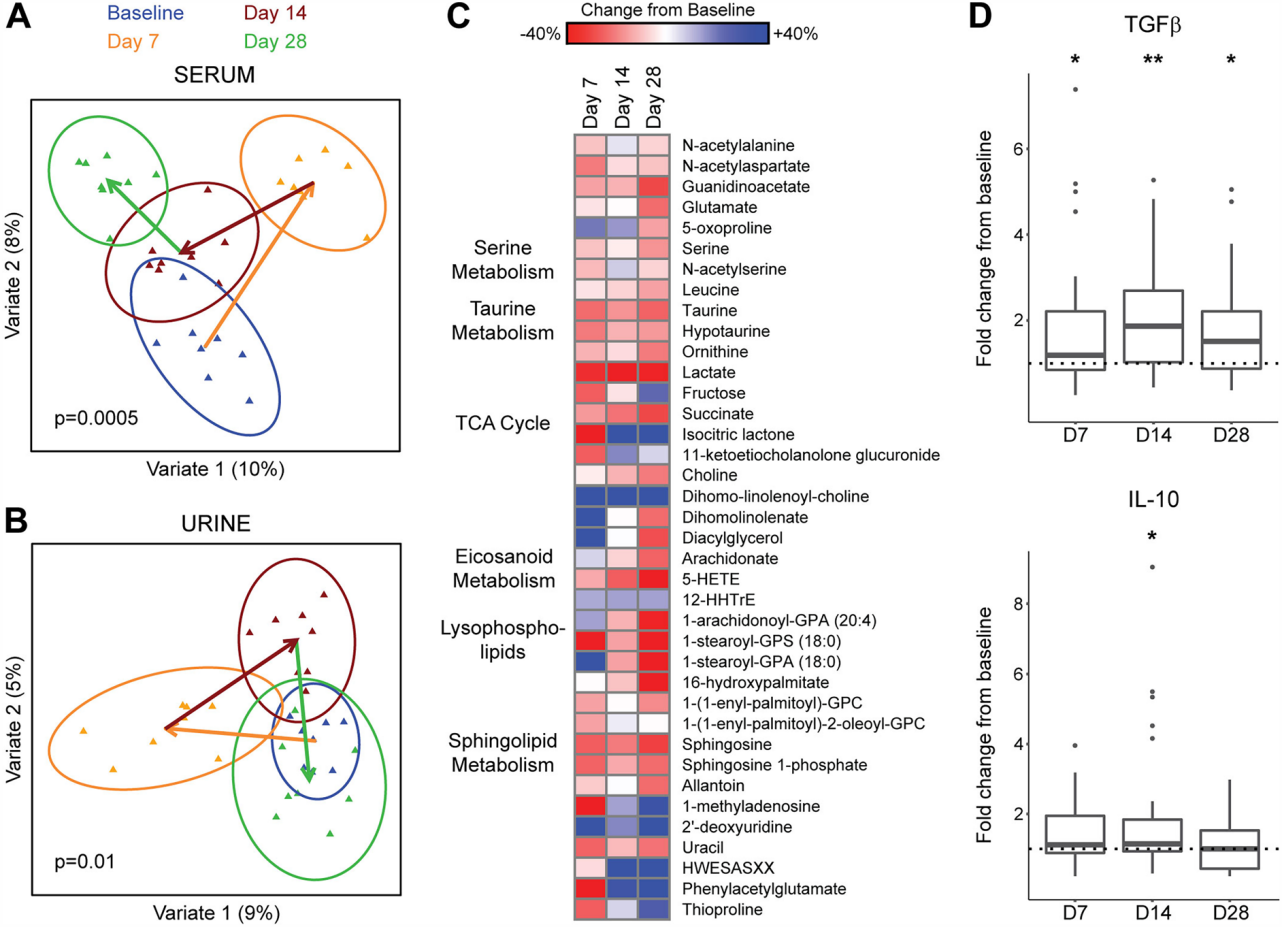
HMO administration alters circulating levels of metabolites and cytokines. (**A, B**) Multilevel sPLS-DA plot visualizing shifts in metabolomics profiles in the serum (**A**) and urine (**B**) across the four time points controlling for subject. Ellipses indicate the 95% confidence region for each time point. Arrows indicate change in centroids at each time point. P-values were calculated by Adonis, adjusting for subject. (**C**) Heat map showing differentially abundant serum metabolites, ordered by metabolic pathway assignment. Color gradient indicates percentage increase or decrease in metabolite relative abundance. (**D**) Boxplots show fold change in measured circulating concentration of TGFβ and IL-10 at each of the study time points compared to baseline. The dotted lines indicate a fold change of 1. *p < 0.05, **p < 0.01.

Serum and urine global metabolomics were performed for the highest dose group to investigate the systemic effects of HMO-Concentrate. Significant shifts in both serum and urine occurred across the four time points (Fig. 6A, B). As had been observed with the intestinal microbiome data, day 7 and day 14 showed distinct profiles from baseline and from one another. Interestingly, the urine metabolome showed reversion to baseline by day 28 whereas the serum metabolome on day 28 further diverged from baseline. Differential abundance testing demonstrated statistically significant changes in 33 serum metabolites at day 28 (Fig. 6C). These changes affected diverse metabolic pathways and included depletion of circulating succinate, lactate, sphingosine, and sphingosine-1-phosphate. Similar trends in these serum metabolites were observed at earlier time points but none reached significance on days 7 and 14. No individual metabolites in the urine achieved statistical significance at any of the time points.

Circulating cytokine levels were measured to assess the effect of HMO treatment on systemic immune status. IL-2, IL-4, IL-5, IL-6, and IL-12 were not detectable in most samples. There were no significant changes from baseline in levels of TNFα or IFNy (data not shown). An increase in TGF-β was observed on Day 7 which persisted on days 14 and 28; there was particularly notable induction in the 18 g dosage group (Fig. 6D). Increased levels of IL-10 were observed on day 14, though no other time point was significantly different from baseline.

### HMO-concentrate induces shifts in cultured fecal microbiota that are not fully recapitulated by mixtures of individual abundant HMOs

**Figure 7:**
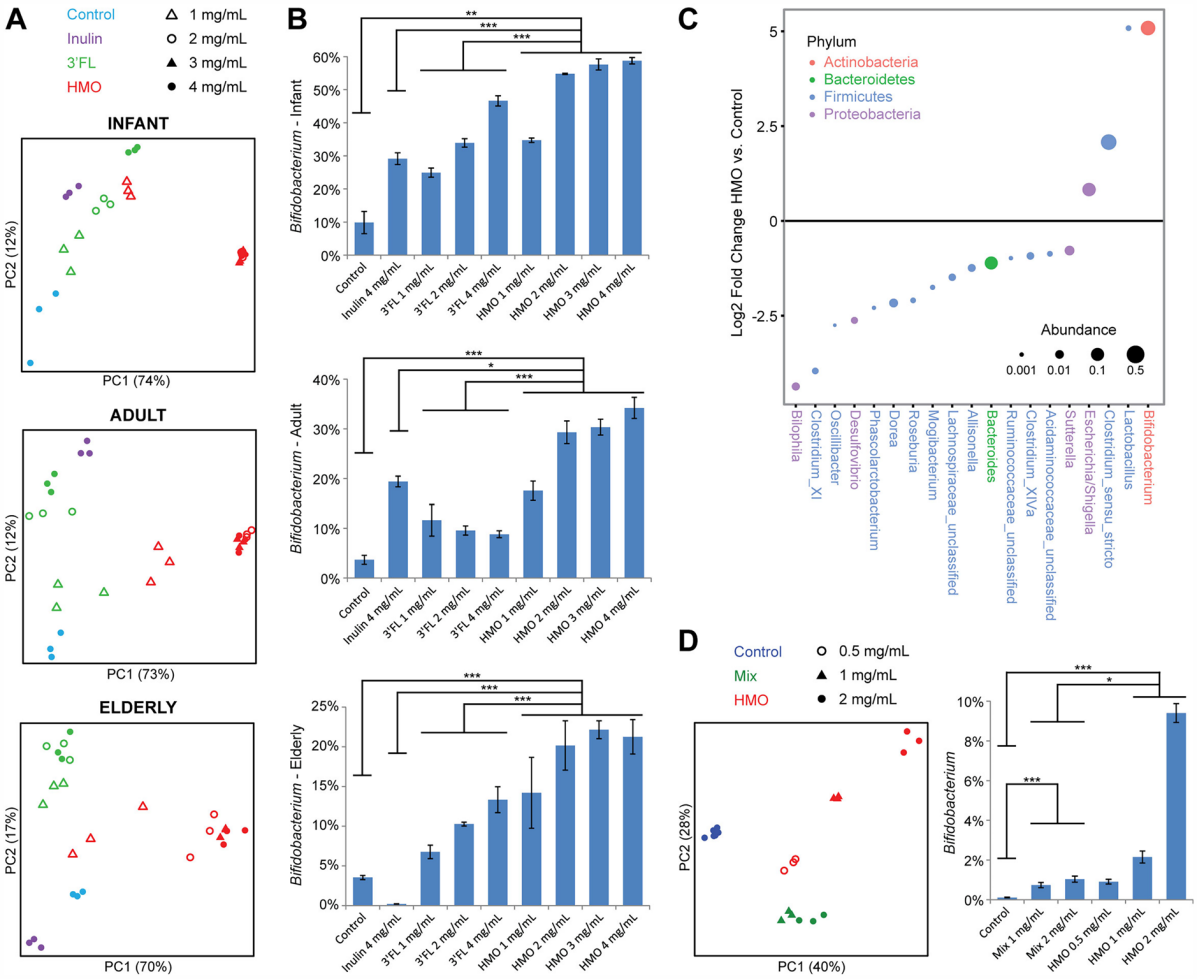
HMO-Concentrate induces *Bifidobacterium* expansion in cultured human fecal microbiota. (**A**) PCoA plots of pooled human fecal microbiota from infants, adults, or elderly donors after 24 h of anaerobic culture with or without varying doses of HMO-Concentrate, 3’-FL, and inulin added to media. (**B**) *Bifidobacterium* abundance is shown for each experimental group (mean + /− SEM). *p < 0.05, **p < 0.005, ***p < 0.001 (**C**) Differentially abundant genera in HMO treated adult and elderly microbiota for 24 h compared to control based on DESeq2 models adjusted for type of microbiota and HMO dosage. (**D**) PCoA plot and *Bifidobacterium* abundance for cultured adult microbiota exposed for 24 h to HMO-Concentrate or a mixture of the top 10 most abundant individual HMOs in the concentrate at their measured concentrations.

Given that HMO-Concentrate could have effects on the epithelium and mucosal immunity that secondarily influence the gut microbiome, we utilized an in vitro strategy to characterize the direct response of the adult intestinal microbiome to HMO-Concentrate. Bioreactors were inoculated with human microbiota derived from stool obtained from adult, elderly, or infant donor fecal samples under anaerobic conditions. Cultures were treated for 24 h with HMO-Concentrate, the most abundant single HMO in the concentrate (3’FL), or a prebiotic control (inulin). While both inulin and 3’FL altered the microbiome, these effects were distinct from that seen with HMOs which showed a dose-dependent alteration of microbiome composition along a principal coordinate explaining 70–74% of variation (Fig. 7A). *Bifidobacterium* expansion was observed after incubation with HMOs in all three microbiota donor groups, in each case to a greater degree than that observed in the inulin or 3’FL groups at comparable dose (Fig. 7B). Differential abundance testing showed *Bifidobacterium* to have the greatest magnitude of change of any taxa with HMO concentrate; other changes with HMO incubation included decreased *Bacteroides* and *Roseburia*, which had been observed in patients treated with HMO at day 7 (Fig. 7C). Short chain fatty acids were measured in the media of anaerobic cultures after 24 h to demonstrate the capacity of adult and elderly microbiota to ferment HMOs. HMO-Concentrate induced greater acetate production in all three donor microbiota age groups compared to 3’FL or inulin (Fig. S5). Interestingly, HMO-Concentrate induced butyrate production by the adult and elderly microbiota but not the infant microbiota. HMO inhibited propionate production relative to 3’FL, inulin, or untreated controls by all three microbiota donor groups.

Additional experiments were undertaken to compare the effect of HMO-Concentrate to that of individual HMOs found within the concentrate. The top ten most abundant HMOs could not recapitulate the effects of the HMO-Concentrate on the microbiome in vitro when added individually to culture media at their measured concentration in the HMO-Concentrate (Fig. S6). Moreover, a mixture of the five most abundant non-sialylated HMOs had only partial effect on microbiome composition compared to HMO-Concentrate and a mixture of the five most abundant sialylated HMOs had no effect. A mixture of the 10 most abundant HMOs at their concentration measured in the HMO-Concentrate was then tested. This mixture showed reduced *Bifidobacterium* expansion compared to an equal dose of the HMO-Concentrate and only a partial effect on microbiome composition, comparable to HMO-Concentrate at a quarter of the dose (Fig. 7D).

## Discussion

This study demonstrated that oral administration of HMO-Concentrate derived from industrially processed, pooled donor human milk permeate is safe, well-tolerated, and can modulate the intestinal microbiome of adults. HMOs induced a robust expansion of *Bifidobacterium*, which is consistent with the known ability of these microbes to effectively utilize HMOs and suggests that sufficient *Bifidobacterium* reservoirs exist in healthy adults to rapidly utilize ingested HMOs. Addition of HMOs to adult intestinal microbiota anaerobic cultures resulted in broadly concordant microbiome shifts as seen in the human study as well as evidence of fermentation to short chain fatty acids. This supports a direct prebiotic effect of HMOs on the microbiome as opposed to indirect effects via the epithelium or mucosal immunity.

The in vitro experiments provide insight into the relative effect of pooled donor HMO-Concentrate compared to individual HMOs and inulin, a common prebiotic that has previously been reported to induce *Bifidobacterium* in adult recipients^41^. Our in vitro data showed *Bifidobacterium* induction in cultured adult microbiota treated with inulin but with a lower effect size than with HMO. However, inulin treatment of cultured elderly microbiota actually triggered *Bifidobacterium* depletion. While it’s unclear whether the different response is attributable to differences in age groups or other sources of variation between the two human fecal donor pools, the results suggest that *Bifidobacterium* induction by inulin is context dependent and that HMOs may be more consistent inducers of *Bifidobacterium*. Also, since HMO only expanded a narrow range of endogenous bacteria consisting primarily of *Bifidobacterium* species, it could be advantageous as a component of *Bifidobacterium*-containing synbiotics as compared to prebiotics such as inulin that are metabolized by a wide range of bacteria and may have unpredictable and even deleterious effects. A recently published paper by Button et al*.* demonstrated enhanced engraftment of *B. infantis* in healthy volunteers when given along with an HMO preparation similar to the HMO-Concentrate in this study^42^. The research described in this paper was chronologically first, and the results contributed to the decision to study engraftment of *B. infantis* given along with HMO in healthy volunteers.

Individual HMOs or defined mixtures of up to 10 highly abundant HMOs failed to fully recapitulate the effects of pooled donor HMO in vitro. This may reflect wide variation across adult *Bifidobacterium* strains in their ability to utilize specific HMO as has been documented in infant strains^43^. Diverse HMO structures, including those in minimal abundance, may be required in HMO product to ensure availability of a substrate for the specific *Bifidobacterium* strains present in each individual adult microbiome. The lack of apparent *Bifidobacterium* species selection with HMO treatment in the human study may be explained by the ability of members of *Bifidobacterium* communities to share HMO milk degradation products due to extracellular HMO degrading enzymes^44,45^. Interestingly, *Collinsella aerofaciens* and *Ruminococcus obeum*—two non-*Bifidobacterium* species associated with the infant gut microbiome and present in the adult gut microbiome—expanded alongside *Bifidobacteirum* in response to HMOs. This suggests that these microbes carry machinery for metabolizing HMOs or are able to share metabolism with *Bifidobacterium* species to expand in response to HMOs.

Increased levels of the SCFAs acetate and butyrate were observed after HMO treatment. While these changes fell just short of significance, the findings are consistent with the in vitro experiments and prior work supporting that infant microbiota convert HMOs to SCFAs^46^. The high variability of fecal SCFA levels in the human study in contrast to the consistent SCFA induction seen in vitro likely reflects increased epithelial uptake/utilization of SCFAs in parallel with increased production, such that fecal levels only loosely correlated with microbial production. Of note, a prior study of 2’FL and LNnT supplementation to healthy adult volunteers for two weeks had also not reported any significant effects on fecal SCFA levels despite *Bifidobacterium* expansion^33^. A potential in vivo correlate of increased local SCFA production is the significant elevation of circulating TGFβ that occurred after 7 days of HMO treatment, with greatest induction in the highest dose group. Butyrate has been reported to be the primary microbial metabolite inducing TGFβ expression by intestinal epithelial cells and is a plausible candidate microbial mediator of altered circulating TGFβ levels^47^.

HMO supplementation also resulted in shifts in the functional capacity of the microbiome including increased levels of bacterial genes involved in antibiotic synthesis and decreased levels of genes involved in antibiotic resistance, biofilm formation, bacterial invasion into epithelial cells, and infection. Taken together, these shifts indicate a microbiome with greater colonization resistance due to endogenous antibiotic production, less pathogenic potential, and increased susceptibility to antibiotic treatment. In conjunction with the known ability of HMO to directly bind pathogens and bacterial toxins, these results suggest that HMO may have anti-infective properties in gastrointestinal infections such as *Clostridioides difficile* colitis^12–14^.

A major unexpected finding of this study was further gut microbiome shifts after cessation of HMO treatment. Instead of reversion to baseline, the gut microbiome underwent a microbial succession in which *Bacteroides* and other Bacteroidetes expanded as *Bifidobacterium* subsided, accompanied by decreased Firmicutes. This resembles the microbial transition seen in infants after cessation of breastfeeding, which is also characterized by *Bacteroides* expansion^48^. Moreover, breastfeeding during infancy is strongly associated with a *Bacteroides*-dominant microbial community state in healthy adults^49^. *Bacteroides* species have been reported to be HMO utilizers and while they did not expand during HMO treatment, they may have been primed by HMO exposure to be well-positioned to expand into the niche vacated by *Bifidobacterium* following cessation of HMO treatment^50^. Reduction in Firmicutes has also previously been reported in a trial of 2’FL and LNnT^33^. Interestingly, increased Bacteroidetes relative to Firmicutes has been suggested to be a marker of reduced obesity propensity in studies comparing obese vs. lean animals and weight-discordant human twins^51,52^. Shifts in the serum metabolome that occurred by day 28—perhaps in response to post-HMO intestinal microbiome changes—could be protective against obesity and metabolic syndrome, in particular reduction in succinate and sphingosine-1-phosphate. Circulating succinate levels are associated with gut microbiome composition and are elevated in patients with high blood pressure, obesity, and diabetes^53^. This clinical association may be mediated by the G-protein coupled receptor SUCNR1, which inhibits lipolysis in adipose tissue and thereby induces retention of fatty acids^53,54^. Elevated circulating sphingosine-1-phosphate has been reported in obesity and may promote insulin resistance through inhibition of insulin signaling via the S1PR2 receptor^55,56^.

While this study has many strengths including longitudinal design, dose titration, multi-omics assessment of microbiome composition/function, and inclusion of in vitro models, there are at least six limitations. First, each dose group had a small sample size, but as this was a pilot study it provides foundational data for future clinical investigation of pooled donor milk HMOs in adult populations. Second, the small sample size precluded assessment of variation by sex and race/ethnicity or lifestyle factors such as long-term dietary habits. We also are unable to extrapolate the study findings to infants, children, or the elderly as the study age range was 18 to 55. Third, the study does not include a placebo arm to assess whether dietary and lifestyle standardization during the 7-day intervention period contributed to some of the findings. However, dose-dependent *Bifidobacterium* expansion when compared to baseline was observed in study subjects, which supports that the effect was specific to HMO treatment. Fourth, there was incomplete concordance between the results of the in vitro experiments and the human study. In particular, prominent *Lactobacillus* expansion was seen in vitro, which is consistent with studies in infants suggesting that *Lactobacillus* utilizes HMOs; however, no change in *Lactobacillus* was seen in the adult human study^28^. This may reflect differences between anaerobic culture conditions and the human intestine as well as the longer duration of HMO exposure in the human study (7 days vs. 24 h). Fifth, this study only included healthy volunteers, so it’s unknown whether HMOs would have similar effects on microbiota associated with intestinal or systemic disease. Sixth, components in HMO-Concentrate besides HMOs may have contributed to the observed microbiome shifts.

In summary, we have demonstrated that HMOs can exert direct effects on adult gut microbiota including marked *Bifidobacterium* expansion, with subsequent ecological shifts as *Bifidobacterium* subsides to baseline following HMO cessation. Pooled donor human milk preparations that include low abundance and naturally occurring HMOs that are not commercially available may be required for maximum effect. This study supports the need for further investigation into the potential therapeutic applications of HMOs as modulators of the intestinal microbiome in diseases associated with dysbiosis or when *Bifidobacterium* expansion is desired. Future directions include follow-up studies with larger numbers of participants, defined cohorts based on demographic traits like age, inclusion of a placebo arm, longer duration of HMO treatment, and study populations with chronic disease.

## Methods

### Study design

This was a phase 1, escalating dose, open-label, single-center study conducted in healthy adult volunteers recruited via community advertisement by WCCT Global, Inc. (ClinicalTrials.gov NCT05516225; registration date 25/08/2022). Inclusion criteria were body mass index (BMI) between 18.5 and 24.9 kg/m^2^, age between 18 and 50 years old, no diagnosed medical conditions, no significant change to diet in the 2 weeks prior to enrollment, and agreement to use contraception for female subjects. Exclusion criteria included lactose intolerance, pregnancy, lactation, and enrollment in another clinical study. Fifty-two subjects were initially screened for study eligibility of which thirty-two were enrolled. Informed consent was obtained from all participants. Subjects were assigned sequentially to one of four dose cohorts, starting with the lowest dose group, in groups of eight based on date of screening and gender requirements (five females and three males to facilitate analysis of the vaginal microbiome). The protocol for this study was reviewed and approved by Alpha IRB (Costa Mesa, CA) before study initiation. This trial was conducted in accordance with the Declaration of Helsinki, International Conference on Harmonization, and Good Clinical Practice guidelines.

### HMO-concentrate

The test product was derived from permeate, a by-product of donor breast milk ultra-filtration during manufacture of commercial donor human milk-based fortifier and ready-to-feed products (Prolacta Bioscience, Duarte, CA). Permeate consists primarily of water, minerals, lactose, and HMO. HMO-Concentrate was generated by subsequent removal of lactose and reduction of minerals from permeate followed by further ultrafiltration, pasteurization and then fill into final containers. The test product was supplied as a frozen liquid, stored at ≤ −20 °C, and thawed on the day of administration. This study sequentially evaluated the following doses of HMO-Concentrate in separate cohorts of 8 subjects each: 1.8 g (0.1 X), 3.6 g (0.2 X), 9 g (0.5 X), and 18 g (1 X) per day, given in three divided doses orally for 7 consecutive days. The doses were based on the daily intake of HMO that a 3.5 kg newborn infant would ingest: 150 mL/kg of mother’s milk per 24 h (3.5 kg × 150 mL/kg × 3.5 mg/mL = 1.8 g). To adjust to an adult dose, a tenfold difference in intestinal surface area between the newborn and the adult was assumed. Consequently, the 1X dose was calculated as 18.0 g of measured oligosaccharides per day.

### Study Visits

The study included 11 visits: Screening Visit (Visit 1) between 4 and 28 days before the start of each study group; Visit 2 on Day -1; Visits 3–9 on Days 1–7; Visit 10 on Day 14; and End of Study Visit (Visit 11) on Day 28. Subjects remained on site at the study center for 7 consecutive days, 8–10 h per day, beginning on Visit 3/Day 1. During this time, subjects received a standard menu and were given the HMO-Concentrate three times daily within 30 min after completion of breakfast, lunch and dinner to take orally. Subjects could only consume food and drinks approved by the study center and were required to ingest the entire dose of the study product to remain enrolled in the study. Subjects were instructed to not ingest probiotics, prebiotics, laxatives, proton pump inhibitors, histamine-2 receptor antagonists, and antacids during the 28 day study period.

### Sample collection

Stool samples were collected on Visits 2, 9, 10, and 11. Subjects defecated into a commode then aliquots were transferred into three Cryovials (one without preservative, two with RNALater for DNA stabilization). Vaginal samples were collected on Visits 2 and 9 by swabbing the left mid lateral wall and right lateral wall, then placing the swab into a sterile micro-centrifuge tube. All samples were stored at −80 °C immediately after collection.

### Safety monitoring

Safety was evaluated by adverse event reporting, clinical laboratory tests, urinalysis, vital signs, electrocardiograms, fecal occult blood, fecal calprotectin, and physical examination. Samples of blood and urine were obtained from subjects on Days 1, 7, 14 and 28 for safety monitoring. Blood was tested for basic metabolic panel, complete blood count, liver panel, total calcium, magnesium, phosphate, and a panel of cytokines including interleukin-2 (IL2), IL4, IL5, IL6, IL10, IL12, tumor necrosis factor α (TNFα), interferon γ (IFNγ), and transforming growth factor β (TGFβ) measured by enzyme-linked immunoassay (ELISA). Urine samples were tested for specific gravity and reducing substance. Stool samples were tested for occult blood and calprotectin.

### 16S rRNA gene sequencing

Microbial DNA was extracted from feces and vaginal swabs using the MO BIO PowerMag® kit (Carlsbad, CA) according to manufacturer’s instructions. The V4 region of the 16S ribosomal RNA gene was amplified and barcoded samples underwent 250 × 2 sequencing on an Illumina MiSeq^57^. Paired-end reads were merged and the resulting sequences were compared to an in-house database using USEARCH. All sequences hitting a unique database entry with an identity ≥ 99% were assigned to a reference-based operational taxonomic unit (OTU). To ensure specificity of the hits, a difference of ≥ 0.25% between the identity of the best hit and the second-best hit was required (e.g. 99.75 vs. 99.5). For each reference-based OTU one of the matching reads was selected as representative and all sequences were mapped by USEARCH against the reference-based OTU representatives to calculate abundances. The remaining sequences were quality filtered and dereplicated with USEARCH. Resulting unique sequences underwent chimera filtering and clustering at 97% by UPARSE (de novo OTU clustering) and a representative consensus sequence per de novo OTU was determined. Representative OTU sequences were assigned taxonomic classification via mothur’s bayesian classifier, trained against the Greengenes database clustered at 99%.

### Shotgun metagenomics

Shotgun metagenomics libraries were prepared using the Nextera XT kit (Illumina) and underwent 150 × 2 sequencing on an Illumina NextSeq. Host sequences were removed with Kraken, and remaining reads were processed with Trimmomatic to trim adapter sequences and low-quality ends (< Q20)^58,59^. MetaPhlAn2 was used to perform taxonomic assignments of non-host metagenomics reads based on an expanded set of approximately 1 million markers (184 + /− 45 for each bacterial species) from > 16,000 reference genomes and > 7,500 unique species^60^. Alpha and beta diversity analyses were performed as described for 16S rRNA gene sequencing. For functional analysis, filtered DNA sequences were mapped against a reference database of all proteins within the Kyoto Encyclopedia of Genes and Genomes (KEGG) databases (version 82.0). The database is composed of 10,012,951 protein sequences extracted from 4,763 genomes. The search of translated DNA sequences was executed using Diamond and hits that spanned ≥  20 amino acids with ≥  80% similarity were collected^61^. In cases where one read matched these criteria against multiple proteins, only the protein or proteins (in the event of a tie) with the maximum bit score were collected.

### Metabolomics

Samples underwent methanol extraction and aliquoting for parallel analysis by four mass spectrometry (MS) pipelines. All methods utilized a Waters ACQUITY ultra-performance liquid chromatography (UPLC) and a Thermo Scientific Q-Exactive high resolution/accurate mass spectrometer interfaced with a heated electrospray ionization (HESI-II) source and Orbitrap mass analyzer operated at 35,000 mass resolution. One aliquot was analyzed using acidic positive ion conditions, chromatographically optimized for more hydrophilic compounds. The extract was gradient-eluted from a C18 column (Waters UPLC BEH C18-2.1 × 100 mm, 1.7 µm) using water and methanol, containing 0.05% perfluoropentanoic acid (PFPA) and 0.1% formic acid (FA). A second aliquot was also analyzed using acidic positive ion conditions, but was chromatographically optimized for more hydrophobic compounds. The extract was gradient eluted from the C18 column using methanol, acetonitrile, water, 0.05% PFPA and 0.01% FA. A third aliquot was analyzed using basic negative ion optimized conditions using a separate dedicated C18 column. The basic extracts were gradient-eluted from the column using methanol and water with 6.5 mM ammonium bicarbonate, pH 8. The fourth aliquot was analyzed via negative ionization following elution from a HILIC column (Waters UPLC BEH Amide 2.1 × 150 mm, 1.7 µm) using a gradient consisting of water and acetonitrile with 10 mM ammonium formate, pH 10.8. The MS analysis alternated between MS and data-dependent MSn scans using dynamic exclusion. The scan range varied slightly by method but covered approximately 70–1000 m/z.

Raw data were extracted, peak-identified, and QC processed using Metabolon’s bioinformatics pipeline. Compounds were identified by comparison to library entries of more than 4500 commercially available purified standards or recurrent unknown entities. Biochemical identifications were based on three criteria: retention index within a narrow retention index window of the proposed identification, accurate mass match to the library + /− 10 ppm, and MS/MS forward and reverse scores. Peaks were quantified using area-under-the-curve. For all analyses, missing values, if any, were imputed with the observed minimum for that particular compound. Raw data was then median scaled prior to further analysis.

### Short chain fatty acids

Fecal samples were analyzed by Metabolon to determine the concentrations of nine short chain fatty acids (SCFAs): acetic acid, propionic acid, lactic acid, isobutyric acid, butyric acid, 2-methylbutyric acid, isovaleric acid, valeric acid, and caproic acid (hexanoic acid). Homogenized samples were spiked with stable labelled internal standards and subjected to protein precipitation with an organic solvent. After centrifugation, the supernatant was derivatized to form the corresponding short chain fatty acid hydrazides which were then injected onto an Agilent 1290/AB Sciex QTrap 5500 LC MS/MS system equipped with a C18 reversed phase UHPLC column. The peak area of the individual analyte product ions was measured against the peak area of the product ions of the corresponding internal standards. Quantitation was performed using a weighted linear least squares regression analysis generated from freshly prepared calibration standards.

Cultured supernatant from in vitro experiments underwent measurement of acetate, propionate, n-butyrate, isobutyrate, and isovalerate as described previously^62^. In brief, exposed material from the i-screen samples was centrifuged (~ 12,000×*g*, 5 min) and the cleared supernatant was filter sterilized (0.45 µm). A mixture of formic acid (20%), methanol, and 2-ethyl butyric acid (internal standard, 2 mg/mL^−1^ in methanol) was added. A 3 µL sample, with a split ratio of 75.0, was injected on a GC-column (ZB-5HT inferno, ID 0.52 mm, film thickness 0.10 µm; Zebron, Phenomenex, Torrance, CA, USA) in a Shimadzu GC-2014 gas chromatograph (Shimadzu Europa GmbH, Duisburg, Germany).

### Human microbiota cultures

The effects of HMO-Concentrate on cultured microbiota were investigated using the i-screen fermentation platform (TNO, Amsterdam, Netherlands). The inoculum consisted of standardized human adult, elderly, or infant donor fecal samples obtained from healthy Caucasian volunteers (6 donors per microbiota type), subject to a European lifestyle and nutrition with no antibiotic usage in the three months prior to sampling. Two sets of experiments were performed. The first included cultures of adult, elderly, and infant microbiota exposed to varying concentrations of HMO-Concentrate, inulin, or 3’FL. The second included cultures of adult microbiota exposed to varying concentrations of HMO-Concentrate, individual HMOs, or defined mixtures of HMOs. Experiments were performed in a closed box with an anaerobic strip (AnaeroGen, Oxoid, Cambridge, UK) inside as described previously^63^. To create the standardized microbiota, pooled stools were grown in a fed-batch fermenter for 40 h. The fermentation medium was based on the standard ileal efflux medium (SIEM) composition that was modified as described previously and adjusted to a pH of 5.8^63^. This standard adult gut microbiota was stored at − 80 °C in 12% glycerol. Before starting the i-screen incubations, the standardized fecal inoculum was incubated in the modified SIEM overnight (37 °C; 300 rpm) using a Whitley A45 anaerobic cabinet (Kentron Microbiology BV, Doetinchem, Netherlands) and an 80% N_2_/10% CO_2_/10% H_2_ gas mixture. HMO and other substrates were then mixed with SIEM and the 1% (v/v) fecal inoculum in each well of a deep-well plate. After 24 h of fermentation, collected samples were directly stored at − 20 °C for subsequent DNA extraction and measurement of SCFAs.

### Statistical analysis

Taxonomic abundance tables from 16S rRNA gene sequencing and shotgun metagenomics underwent alpha diversity analysis (Chao1, Shannon) in QIIME 1.9.1^64^. Comparison of microbial composition and gene content across samples was performed using the square root of the Jensen-Shannon divergence and visualization by principal coordinates analysis (PCoA). Significance of differences across time points, adjusting for subject and dose group, was assessed using permutational multivariate analysis of variance (PERMANOVA) with significance determined by 100,000 permutations^65^. Multilevel sparse partial least squares discriminant analysis (sPLS-DA) was performed in mixOmics to provide supervised visualization—adjusting for subject effects—of differences in microbial composition or gene content based upon features that most discriminate the variable of interest^66^. Differential abundance was tested using multivariate negative binomial models implemented in DESeq2^67,68^. Q-values were calculated with the Benjamini–Hochberg procedure to control the false discovery rate.

Metabolomics data were visualized by sPLS-DA. Significance of differences in metabolomics profiles was determined by PERMANOVA based upon the square root of the Jensen-Shannon divergence. Differentially abundant metabolites were identified using general linear models implemented in the limma package with significance assessed by empirical Bayes moderated t-statistics^69,70^. Q-values were calculated with the Benjamini–Hochberg procedure to control the false discovery rate. Metabolic pathway assignments of differential metabolites were based upon Metabolon curated annotations.

Significance of longitudinal changes in alpha diversity metrics, SCFAs, fecal calprotectin, C-reactive protein, and serum cytokines was determined by the Friedman test with the post-hoc Conover test. Significance of differences in short chain fatty acid levels in supernatants from in vitro experiments was determined by two-way analysis of covariance (ANCOVA) with dose as a covariate.

### Supplementary Information

Supplementary Information.

## Data Availability

The 16S rRNA and shotgun metagenomics sequencing data are available in the NCBI Bioproject repository, PRJNA612630 (https://www.ncbi.nlm.nih.gov/bioproject/612630).
