# Supplementary material for: Human milk oligosaccharides modulate the intestinal microbiome of healthy adults
Source: Sci Rep. 2023 Aug 31;13:14308. doi: 10.1038/s41598-023-41040-5 (PMC10471580; doi:10.1038/s41598-023-41040-5)
Supplement: Supplementary file 1 — Supplementary Information. [file 41598_2023_41040_MOESM1_ESM.docx]

Supplementary Materials for

**Human Milk Oligosaccharides Modulate the Intestinal Microbiome of Healthy Adults**

Jonathan P. Jacobs,^*^ Martin L. Lee, David Rechtman, Adam Sun, Chloe Autran, Victoria Niklas

*Corresponding author. Email: JJacobs@mednet.ucla.edu

**This file includes:**

Figs. S1 to S6

Table S1

**
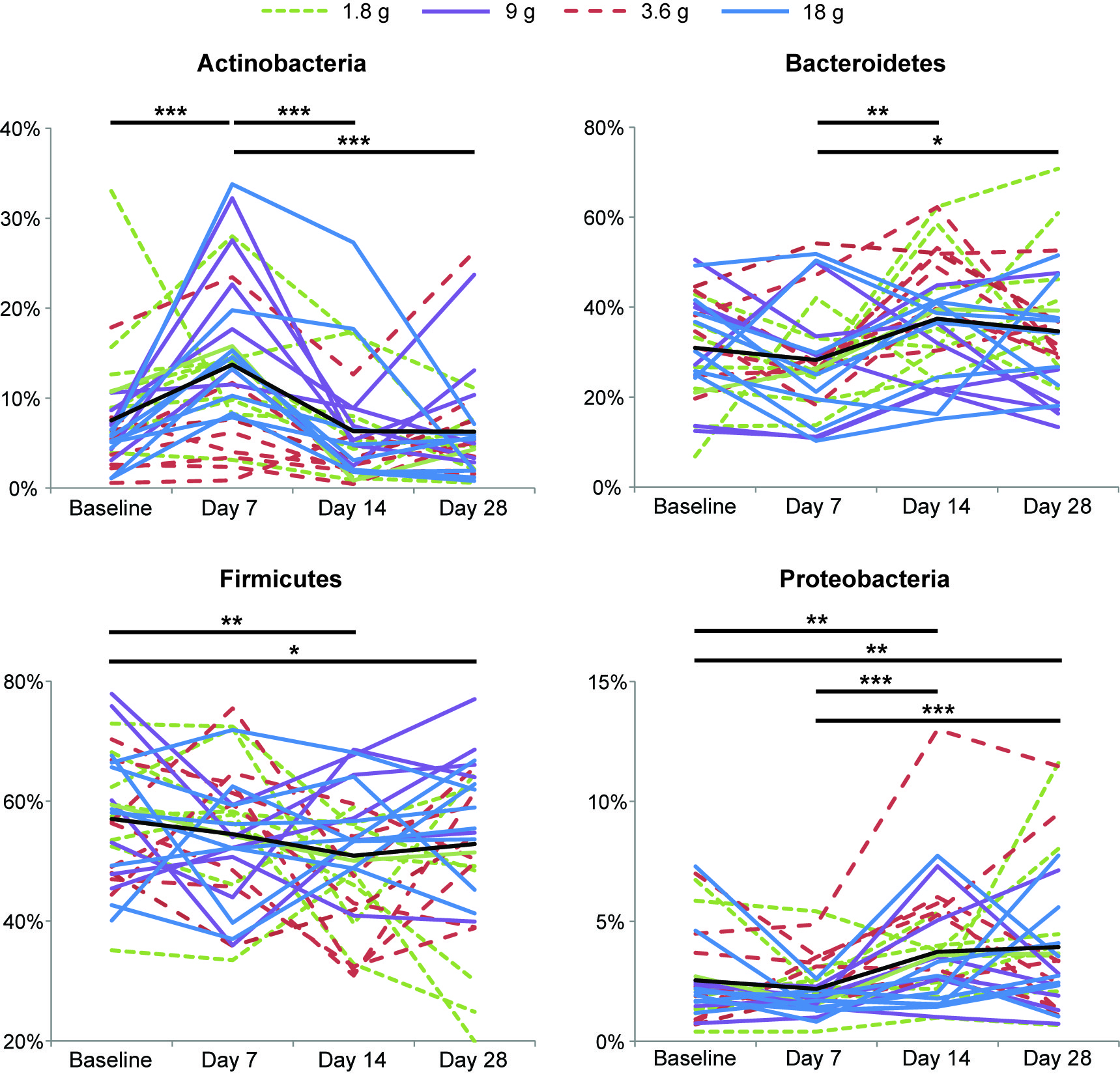
**

**Figure S1. Relative abundances of differentially abundant phyla across the four study time points.** Each line represents one subject. Line color and dash pattern indicate dose group. *p<0.05, **p<0.01, ***p<0.001

**
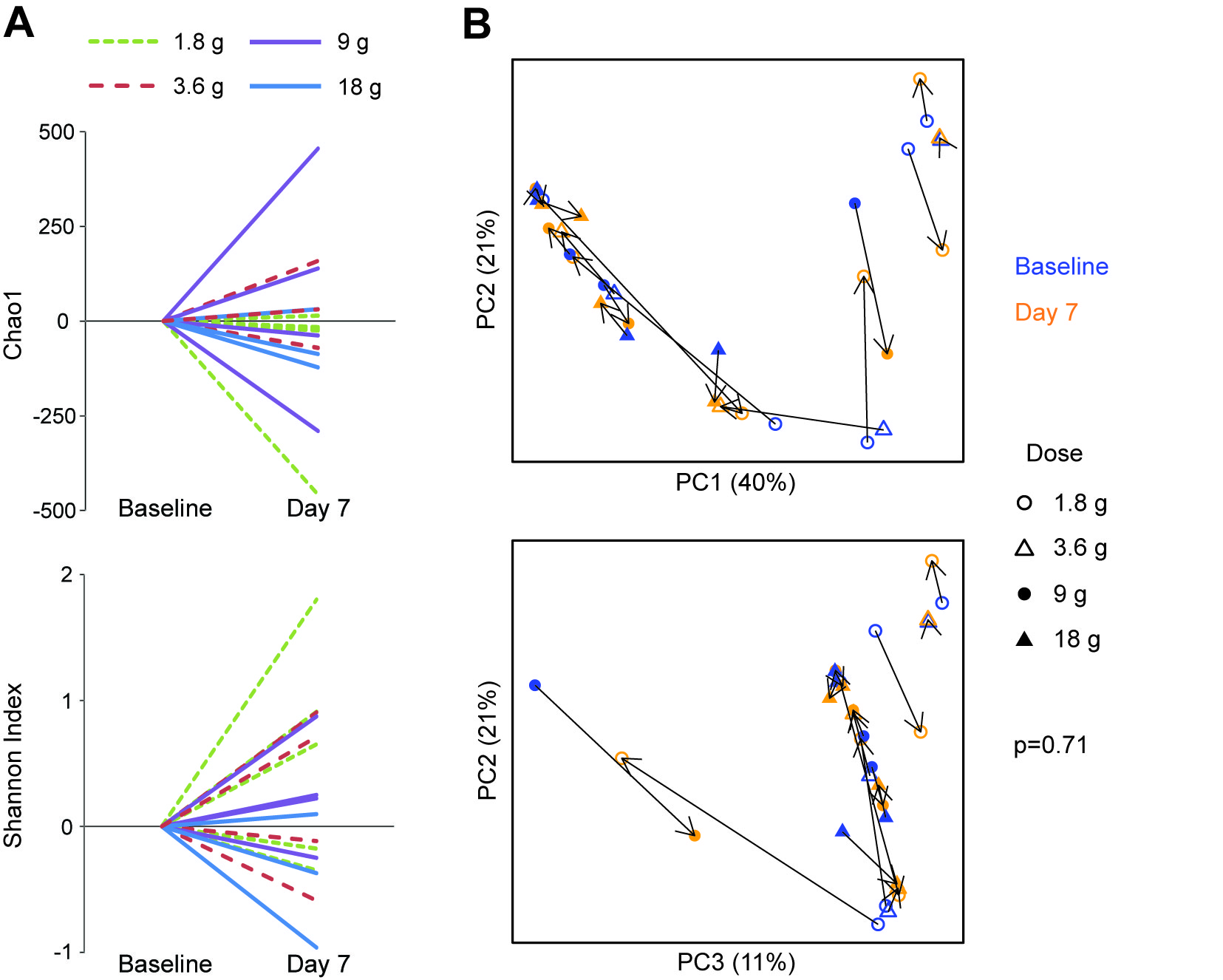
**

**Figure S2. HMO administration did not alter the vaginal microbiome.** (**A**) Changes in alpha diversity (measured by Chao1 and Shannon index) from baseline to day 7 are shown. Each line represents one subject. Line color and dash pattern indicate dose group. (**B**) PCoA plots depicting vaginal microbiome composition between baseline and day 7. Each dot represents one subject, with lines connecting samples collected from each subject. Color represents time point and symbol represents dose group.


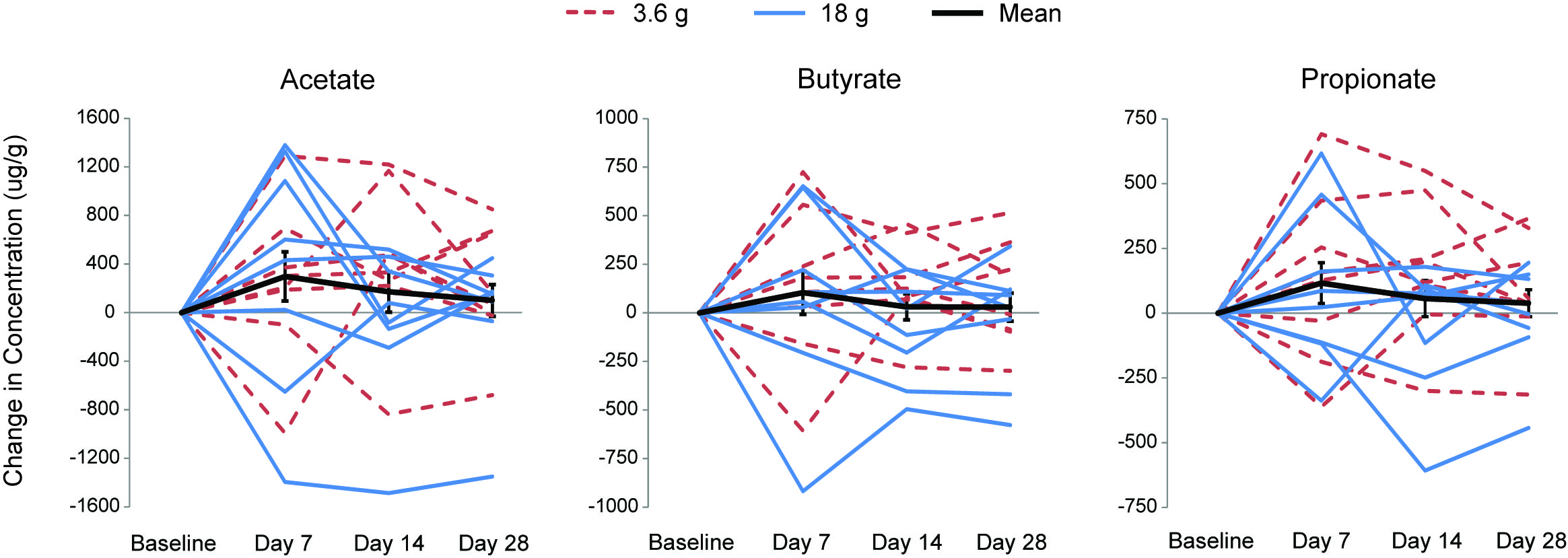


**Figure S3. Trend towards increased short chain fatty acids after 7 days of HMO treatment.** Change in concentration (μg/g feces) from baseline of acetate, butyrate, and proprionate is shown for subjects in the 3.6 g and 18 g dose groups. Each line represents one individual.


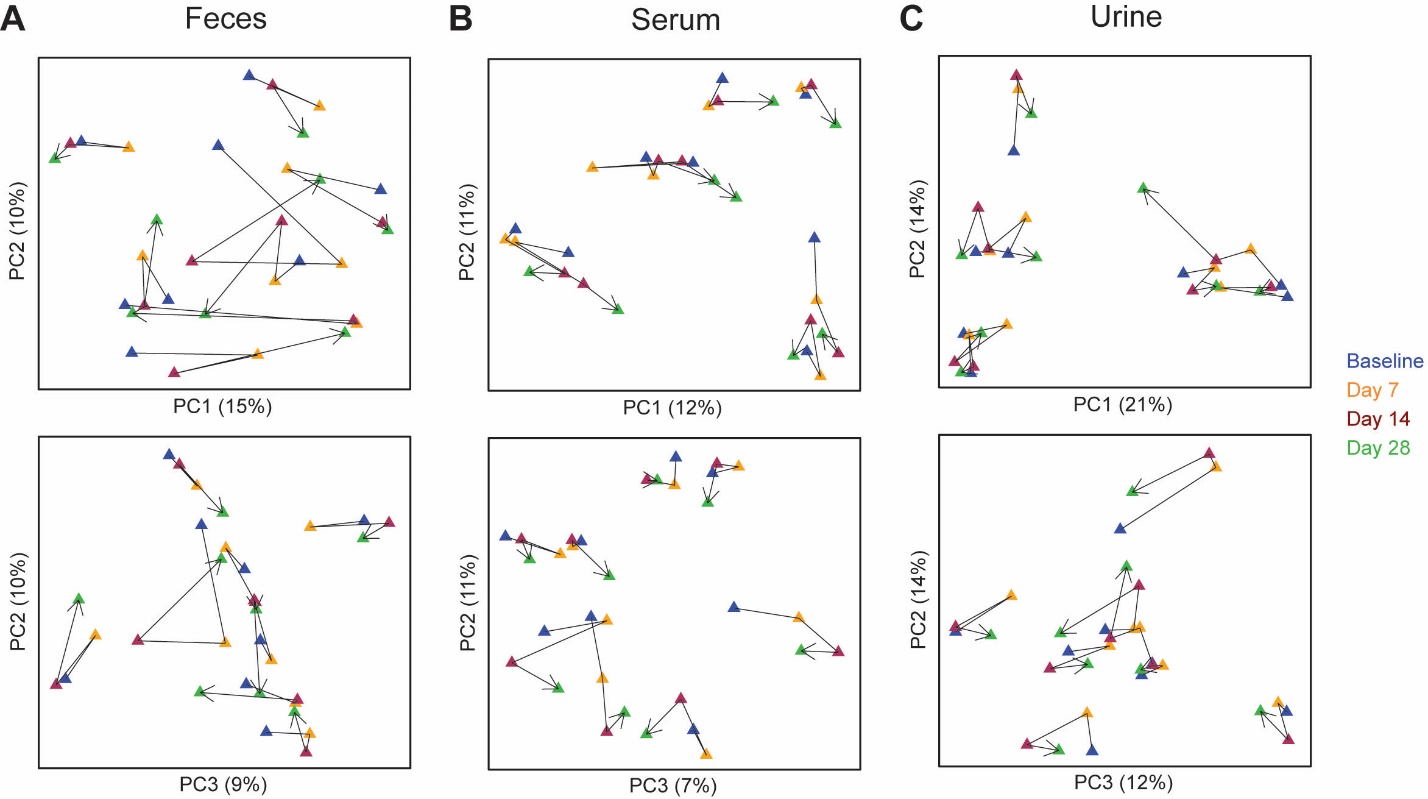


**Figure S4. Subject-specific metabolomics profiles in the feces, serum, and urine across study time points.** PCoA plots depicting metabolomics profiles in the feces, serum, and urine of subjects in the 18 g dose group across the four study time points. The two plots for each sample type show the first three principal coordinates and the percent variation that they explain. Each dot represents one sample, with lines connecting samples collected from each subject.


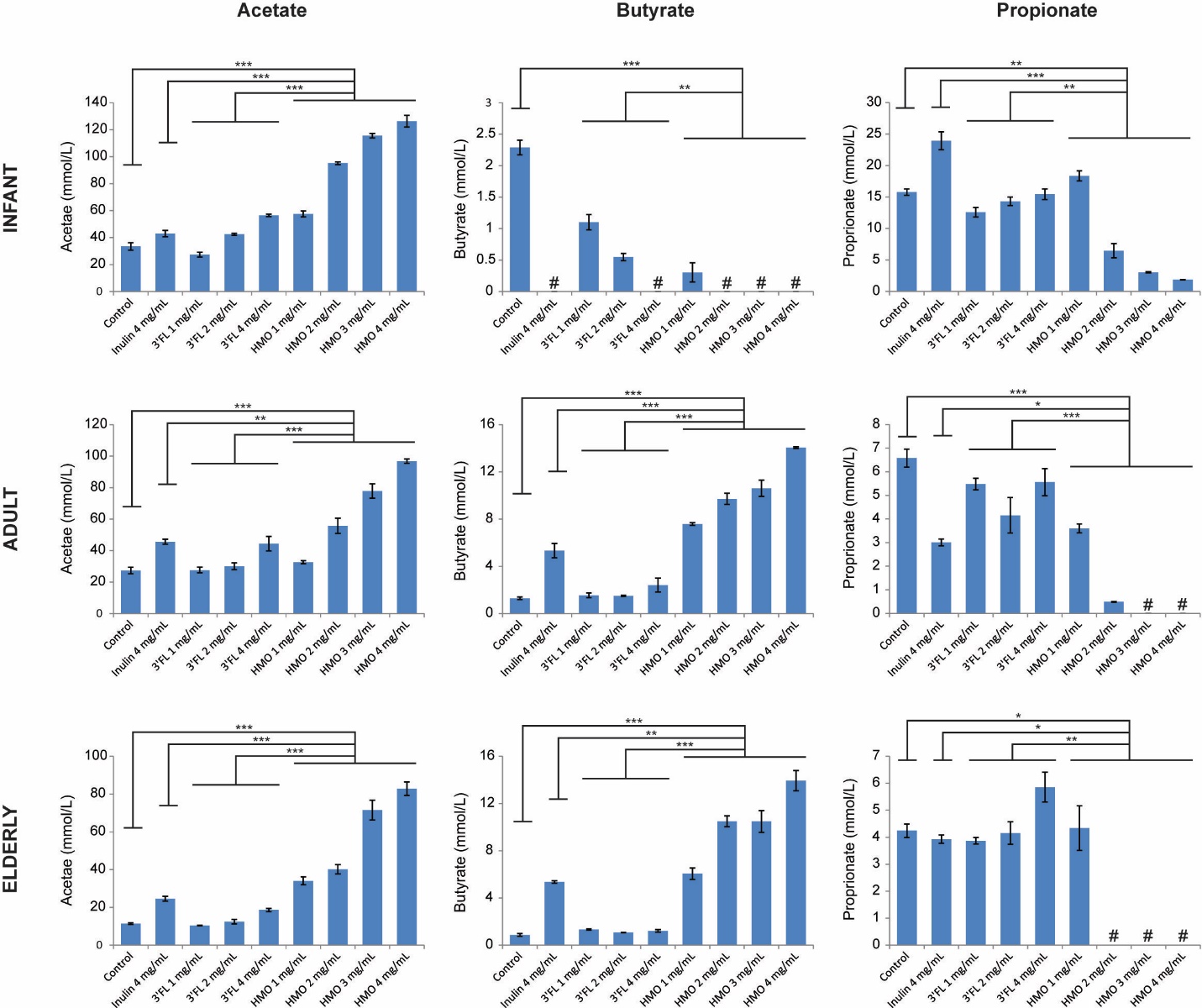


**Figure S5. Pooled donor HMO induces acetate and butyrate production and inhibits propionate production by cultured human adult and elderly fecal microbiota.** Acetate, butyrate, and propionate concentrations were measured in the media of cultured infant, adult, and elderly microbiota after 24 hours with or without pooled donor HMO, 3’-FL, or inulin. # indicates that the short chain fatty acid was undetectable in all samples. Significance determined by two-way ANOVA (models included treatment and dose). *p<0.05, **p<0.005, ***p<0.001


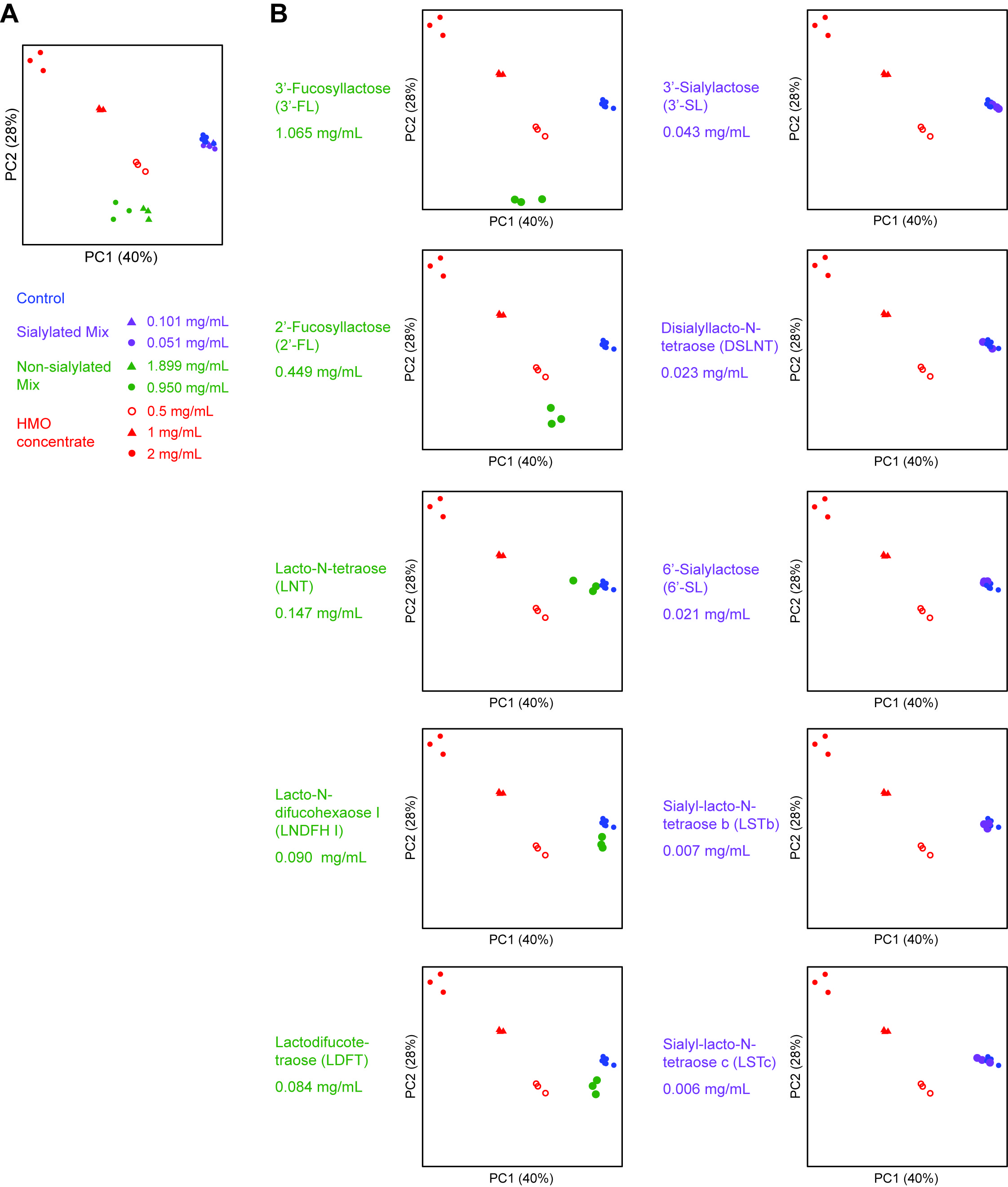


**Figure S6. Comparison of the effects of sialylated and non-sialylated HMOs on cultured human fecal microbiota.** PCoA plots of cultured adult human fecal microbiota after 24 hours of anaerobic culture with or without various HMO preparations. (**A**) A mixture of the top 5 most abundant sialylated HMOs and a mixture of the top 5 most abundant non-sialylated HMOs at their concentrations in HMO-Concentrate were compared to HMO-Concentrate itself. (**B**) The top 10 most abundant individual HMOs in the HMO-Concentrate were added to cultured fecal microbiota at their measured concentration in HMO-Concentrate.

|  | **1.8 g**  **(N = 8)** | **3.6 g**  **(N = 8)** | **9 g**  **(N = 8)** | **18 g**  **(N = 8)** | **Total**  **(N = 32)** |
| --- | --- | --- | --- | --- | --- |
| **Age** | | | | | |
| Mean (SD) | 30 (5) | 30 (7) | 33 (4) | 31 (7) | 31 (8) |
| Median (IQR) | 29 (7) | 28 (8) | 33 (5) | 31 (5) | 31 (8) |
| **Sex** | | | | | |
| Female – n (%) | 5 (63%) | 5 (63%) | 5 (63%) | 5 (63%) | 20 (63%) |
| Male – n (%) | 3 (38%) | 3 (38%) | 3 (38%) | 3 (38%) | 12 (38%) |

**Table S1. Demographic characteristics by dose group.** SD = standard deviation, IQR = interquartile range
